# Five-Year Safety and Effectiveness of Paclitaxel Drug-Coated Balloons Alone or With Provisional Bare Metal Stenting for Real-World Femoropopliteal Lesions: IN.PACT Global Study Subgroup Analysis

**DOI:** 10.1161/CIRCINTERVENTIONS.123.013084

**Published:** 2024-02-13

**Authors:** Gary M. Ansel, Marianne Brodmann, Krishna J. Rocha-Singh, Jeremiah S. Menk, Thomas Zeller

**Affiliations:** 1 Department of Medicine, University of Toledo, OH (G.M.A.).; 2 Healthcare Insights, LLC, Boston, MA (G.M.A.).; 3 Division of Angiology, Medical University, Graz, Austria (M.B.).; 4 Department of Cardiology, Prairie Heart Institute, St. John’s Hospital, Springfield, IL (K.J.R.-S.).; 5 Peripheral Vascular Health, Medtronic, Minneapolis, MN (J.S.M.).; 6 Universitäts-Herzzentrum Freiburg, Kardiologie und Angiologie II, Bad Krozingen, Germany (T.Z.).

**Keywords:** angioplasty, chronic limb-threatening ischemia, intermittent claudication, paclitaxel, peripheral artery disease, stents

## Abstract

**BACKGROUND:**

The treatment of complex infra-inguinal disease with drug-coated balloons (DCBs) is associated with a significant number of patients undergoing provisional stenting to treat a suboptimal result. To determine the potential long-term impact of DCB treatment with provisional bare metal stenting in complex lesions in real-world patients, a post hoc analysis was performed on data from the IN.PACT Global Study (The IN.PACT Global Clinical Study for the Treatment of Comprehensive Superficial Femoral and/or Popliteal Artery Lesions Using the IN.PACT Admiral Drug-Eluting Balloon). Five-year outcomes were compared between participants who were stented after DCB treatment versus those treated with DCB alone.

**METHODS:**

The IN.PACT Global Study enrolled 1535 participants with intermittent claudication and/or ischemic rest pain caused by femoropopliteal lesions; 1397 patients were included in this subgroup analysis (353 stented and 1044 nonstented). Effectiveness was assessed as freedom from clinically driven target lesion revascularization through 60 months. The primary safety composite end point was defined as freedom from device- and procedure-related death through 30 days, and freedom from major target limb amputation and clinically driven target vessel revascularization through 60 months.

**RESULTS:**

Lesions in the stented group were longer (15.37 versus 10.98 cm; *P*<0.001) and had more total occlusions (54.7% versus 28.6%; *P*<0.001) compared with the nonstented group. The 5-year Kaplan-Meier estimated freedom from clinically driven target lesion revascularization was similar between groups (66.8% stented versus 70.0% nonstented group, log-rank *P*=0.22). The safety composite end point was achieved in 64.5% stented versus 68.2% nonstented participants (log-rank *P*=0.19) as estimated by the Kaplan-Meier method. No significant difference was observed in the cumulative incidence of major adverse events (49.1% stented versus 45.0% nonstented; log-rank *P*=0.17), including all-cause death (19.6% stented versus 19.3% nonstented, log-rank *P*=0.99).

**CONCLUSIONS:**

In this real-world study, revascularization of complex femoropopliteal artery lesions with DCB angioplasty alone or DCB followed by provisional bare metal stenting in certain lesions achieved comparable long-term safety and clinical effectiveness.

**REGISTRATION:**

URL: https://www.clinicaltrials.gov; Unique identifier: NCT01609296.

**WHAT IS KNOWN**

- Drug-coated balloons (DCBs) have been associated with a decreased need for reinterventions compared with percutaneous transluminal angioplasty.
- However, complex cases often require provisional stenting to treat residual stenosis or flow-limiting dissections.
- The need for bare metal stenting after DCB angioplasty increases with increasing lesion complexity, defined by lesion length and the presence of total occlusion and circumferential calcium.

**WHAT THE STUDY ADDS**

- This post hoc analysis of data from the IN.PACT Global Study showed that treatment of femoropopliteal complex lesions with DCB angioplasty alone or DCB angioplasty with bare metal provisional stenting in certain lesions is safe with good long-term clinical outcomes as demonstrated by similar low reintervention rates and a low incidence of major amputation and all-cause mortality.
- The results also showed that when provisional stenting is required for flow-limiting dissection or residual stenosis after DCB, stenting was performed predominantly with spot or partial stenting, thus avoiding a full-metal jacket.

**See Editorial by Rymer and Gutierrez**

Randomized controlled trials (RCTs) have consistently demonstrated superior clinical efficacy of drug-coated balloons (DCBs) over standard percutaneous transluminal angioplasty for femoropopliteal peripheral artery disease.^1^ Some of the DCB RCTs have now reported 5-year outcomes and the results continued to show sustained benefits of DCB.^2–5^ As more clinical evidence continues to accumulate in favor of DCB, there is also an increased use of DCB in daily practice for femoropopliteal artery disease. In a recent report from the Vascular Quality Initiative registry, of the 19 672 femoropopliteal endovascular procedures reported, 37% were treated with a DCB in the overall cohort and 42% were treated with a DCB in lesions 10.0 to 19.9 cm long.^6^

There is a common cry for the leave nothing behind treatment of vascular disease, and while DCBs appear to answer this desire, residual stenosis and flow-limiting dissection remain an issue in real-world complex lesions.^7–9^ Provisional stenting is an important tool to mitigate the effects of significant dissection and elastic recoil in complex disease.^10^ If provisional stent placement is indicated, the stented lesion length is frequently shorter than the total lesion length.^11^ The XLPAD registry (Excellence in Peripheral Artery Disease) demonstrated that provisional stenting was safe and effective.^12^ However, longer term sequelae, such as the patency of bare metal stents placed after DCB to optimize a final result, have not been reported.

The IN.PACT Global Study (The IN.PACT Global Clinical Study for the Treatment of Comprehensive Superficial Femoral and/or Popliteal Artery Lesions Using the IN.PACT Admiral Drug-Eluting Balloon) was a prospective, multicenter, single-arm study of the paclitaxel IN.PACT Admiral DCB (Medtronic) in real-world patients with a range of complex lesion types, such as long lesions and chronic total occlusions (CTOs)^.11, 13-15^ In a predefined imaging cohort analysis of the IN.PACT Global Study, participants with lesions ≥15 cm treated with DCB with or without provisional stenting had primary patency rates of 89.0% versus 92.5%, respectively, at 1-year postprocedure.^11^ Similar promising 1-year outcomes were found in a predefined imaging cohort analysis of participants treated with DCB for CTO (primary patency 88.4% in stented patients, and 82.7% in nonstented participants).^15^ Further, in the IN.PACT Global full clinical cohort analysis, freedom from clinically driven target lesion revascularization (CD-TLR) through 2 years was similar between groups treated with DCB with or without provisional stenting (80.8% stented and 83.9% nonstented).^14^

To evaluate the long-term effects of provisional stenting in real-world patients with complex femoropopliteal lesions, we performed a post hoc analysis of data from the full clinical cohort of the IN.PACT Global Study. Five-year clinical outcomes were compared between patients who received a bare metal provisional stent after DCB angioplasty versus those who were treated with DCB alone.

## METHODS

### Data Sharing

To minimize the possibility of unintentionally sharing information that can be used to reidentify private information, anonymized data, and materials may be made available to qualified researchers trained in human subject confidentiality protocols. Requests should be sent to the corresponding author and require approval by the study sponsor.

### Study Design and Procedure

**Figure 1.**
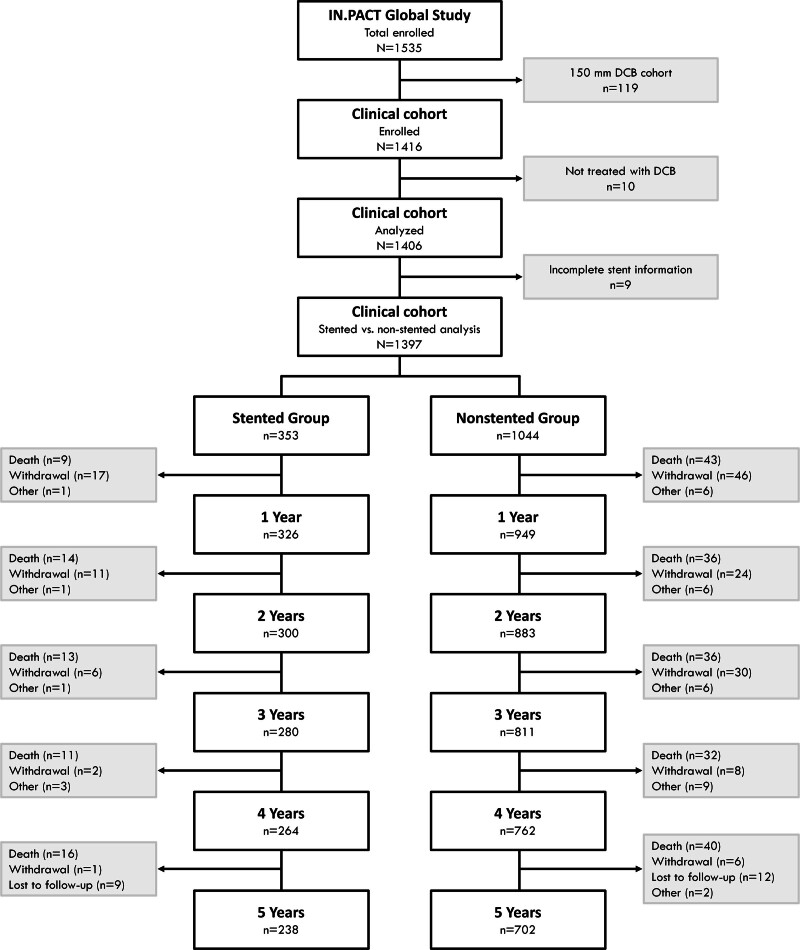
**Participant flow through 5 y in the IN.PACT Global Study (The IN.PACT Global Clinical Study for the Treatment of Comprehensive Superficial Femoral and/or Popliteal Artery Lesions Using the IN.PACT Admiral Drug-Eluting Balloon) stented and nonstented groups.** Annual follow-up windows were 360±60, 720±60, 1080±60, 1440±60, and 1800±60 days. N is the number of participants eligible for analysis at each time point. “Other” refers to exit from the study for reasons other than death, withdrawal, or lost to follow up. DCB indicates drug-coated balloon.

The IN.PACT Global Study was a prospective, multicenter, international, single-arm clinical study to assess the safety and effectiveness of a paclitaxel DCB for the treatment of real-world patients with atherosclerotic disease of the femoropopliteal artery. Participants with intermittent claudication and/or ischemic rest pain (Rutherford clinical categories 2–4) were enrolled across 64 sites in 26 countries from Europe, the Middle East, Asia, North Africa, Australia, Canada, and Latin America from 2012 to 2014. Participants were followed for a total of 60 months (Figure 1), with hospital visits at 6, 12, 24, and 36 months and telephone follow-ups at 30 days, 48 months, and 60 months. To verify safety information, investigational sites were also asked to obtain vital status from study participants who withdrew or were lost to follow-up. These vital status results are labeled as such when those additional data are included. Details of the study design have been reported previously^11,14^ and are described in the Supplemental Methods.

All participants were treated with the IN.PACT Admiral DCB. Provisional bare metal stenting was permitted at the discretion of the operator. The reasons for stent placement were visually estimated residual stenosis ≥50%, translesion gradient >10 mm Hg, or flow-limiting dissection. The present post hoc subgroup analysis was performed to compare 60-month outcomes between participants who received a provisional bare metal stent after DCB treatment (stented group, n=353) and those treated with DCB alone (n=1044).

An independent clinical events committee (Syntactx, New York, NY) reviewed and adjudicated all major adverse events including death, major target limb amputation, clinically driven target vessel revascularization (CD-TVR), CD-TLR, and thrombosis at the target lesion site. The study protocol was reviewed and approved by the institutional review board or ethics committee at each study site, and all participants provided written informed consent before enrollment. The study was conducted in accordance with the Declaration of Helsinki, good clinical practice guidelines, and applicable laws as specified by all relevant governmental bodies. The study is registered URL: https://www.clinicaltrials.gov; Unique identifier: NCT01609296.

### Outcome Assessments

Effectiveness end points were freedom from CD-TLR and the cumulative incidence of CD-TLR within 5 years. In addition, the restricted mean survival time (the expected time to the first CD-TLR up to 5 years postindex procedure) was computed. The 5-year primary safety composite end point was defined as freedom from device- and procedure-related death through 30 days, and freedom from major target limb amputation and CD-TVR through 60 months. Other safety end points included the cumulative incidence of major adverse events (all-cause death, major target limb amputation, CD-TVR, and thrombosis at the target lesion), any target lesion revascularizations, and any target vessel revascularizations within 60 months. Other assessments included device success, procedural success, and clinical success and definitions are provided in Supplemental Methods.

### Statistical Analysis

Baseline demographics and clinical characteristics were assessed on a participant basis, lesion characteristics on a lesion basis, and procedural characteristics on a procedure basis. Continuous variables are summarized as mean±SD, and dichotomous or categorical variables as percentages and counts. Continuous variables were compared with the independent *t* test, and dichotomous and categorical variables using Fisher exact test or Cochran-Mantel-Haenszel modified ridit scores, respectively. The Kaplan-Meier method was used to evaluate time-to-event data over the 60-month follow-up period and was performed as a participant-level analysis using the first event within a participant when multiple lesions were treated. Furthermore, to evaluate the incidence of reintervention in the presence of the competing risk for death, Gray’s test was computed and the cumulative incidence was estimated. Both survival estimates and cumulative incidence are reported along with 95% CIs that were derived using the log-log method. The restricted mean survival time with a time horizon of 1800 days and 95% CI was computed for CD-TLR. The restricted mean survival time is the average time to an event within a fixed time period and corresponds to the area under the survival curve from the start of follow-up to the fixed time point. It incorporates participants with events, censoring, and those with complete follow-up through the time period without an event. No imputation was performed for missing baseline values. No adjustment to the significance level was made for multiple comparisons. The study may be underpowered to detect a difference between subgroups. All results were exploratory, and *P* values were unadjusted. Multivariable analyses based on Fine-Gray and Cox proportional regression models were conducted for CD-TLR and all-cause death, respectively. Detailed methods of these analyses are described in Supplemental Methods. Statistical analyses were performed with SAS version 9.4 (SAS Institute, Cary, NC).

## RESULTS

### Participant Flow, Baseline Characteristics, and Stenting

Participant flow through 5 years is shown in Figure 1. The IN.PACT Global Study enrolled 1535 participants, of which 1416 were in the clinical cohort.^13^ Ten participants were not treated with a DCB and 9 participants had incomplete stent information; they were excluded from the analysis. Of those in the analysis, 353 participants received at least 1 provisional bare metal stent during the index procedure and were assigned to the stented group (25.3%), and 1044 participants (74.7%) were treated with DCB alone and assigned to the nonstented group.

Baseline demographic and clinical characteristics were generally similar between groups, with the exceptions of insulin-dependent diabetes, carotid artery disease, and previous peripheral revascularization, which were less frequently observed in the stented group (Table 1).

**Table 1.**
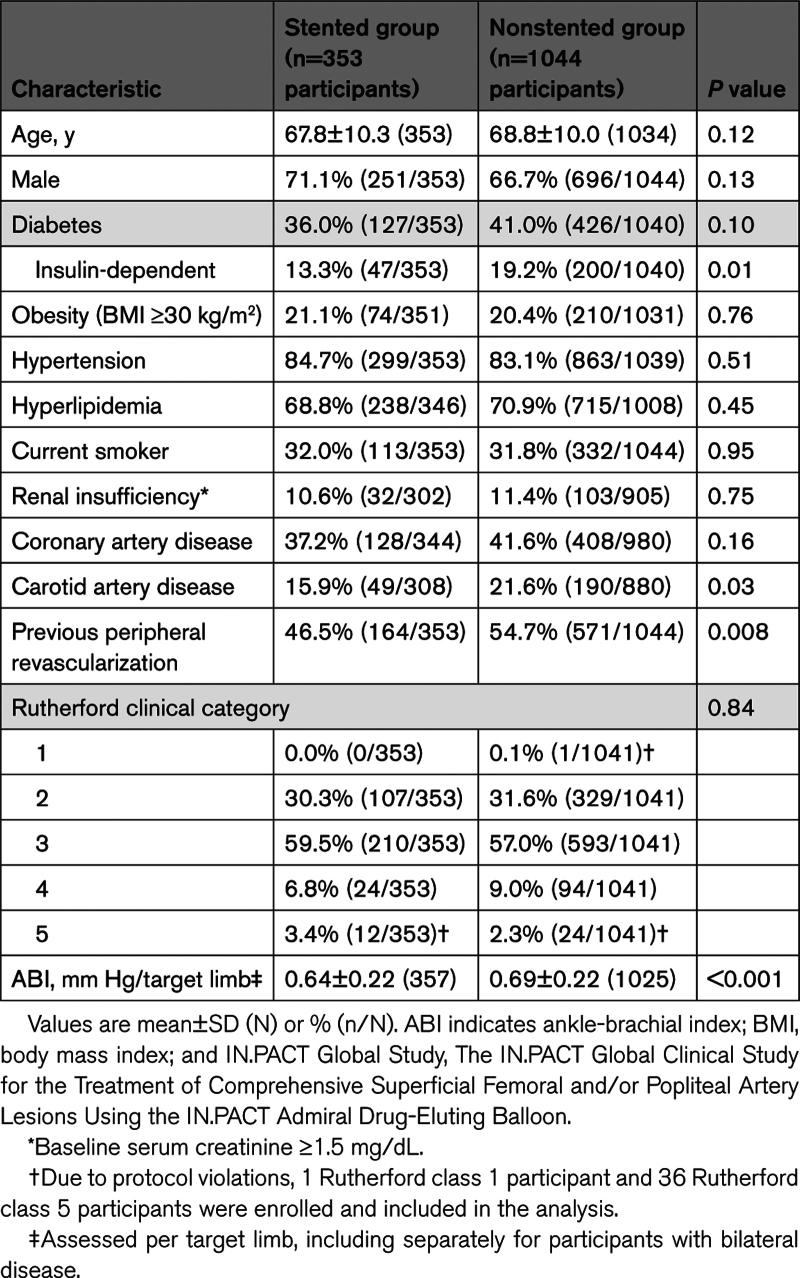
Baseline Demographics and Clinical Characteristics of the IN.PACT Global Study Stented and Nonstented Groups

Overall, participants in the stented group had more complex lesions compared with the nonstented group (Table 2). Lesions in the stented group were longer (mean length, 15.4 versus 11.0 cm nonstented; *P*<0.001) and more stenotic (92.1% versus 87.6% nonstented; *P*<0.001), with a higher frequency of total occlusion (54.7% versus 28.6% nonstented; *P*<0.001) and calcification (73.8% versus 66.7% nonstented; *P*=0.005) including severe calcification (14.7% versus 8.7%; *P*<0.001).

**Table 2.**
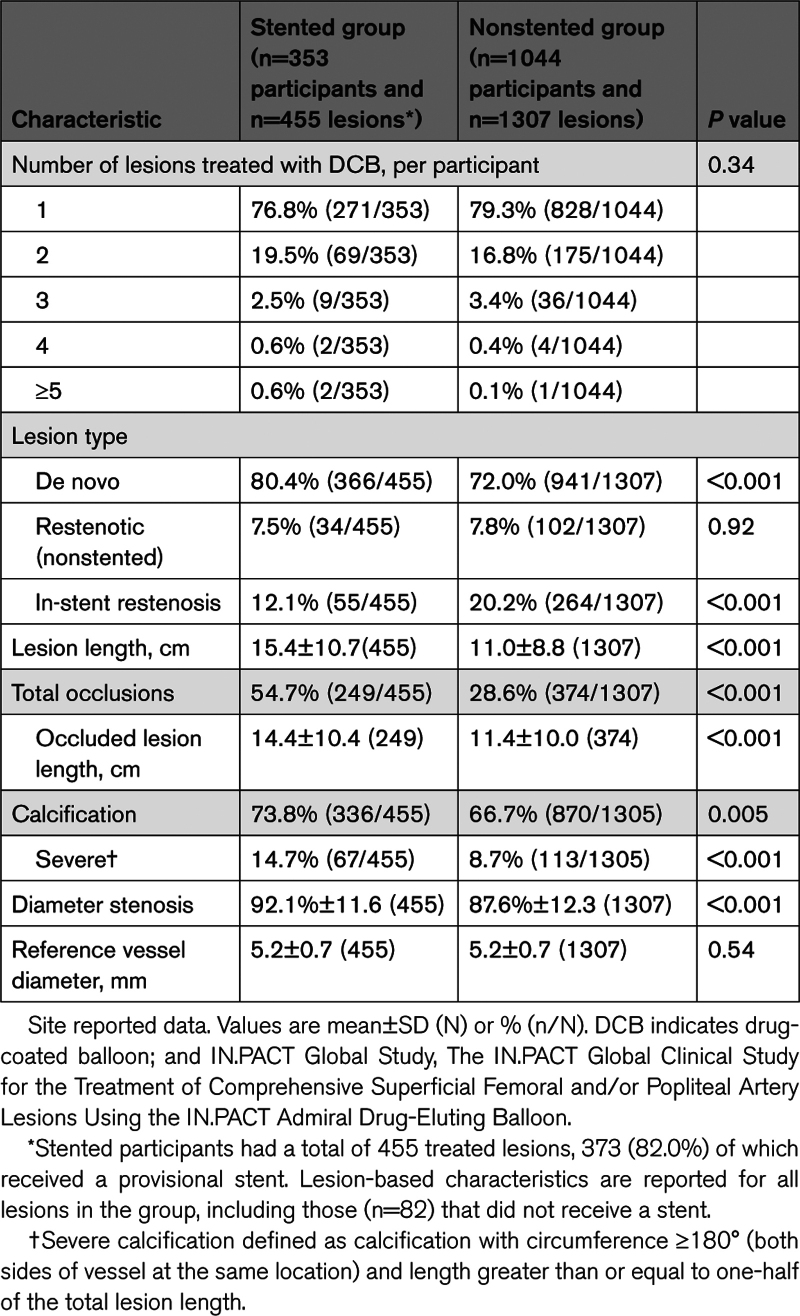
Baseline Lesion Characteristics of the IN.PACT Global Study Stented and Nonstented Groups

Stenting characteristics are reported in Table 3.^16^ Participants in the stented group had a total of 455 lesions and most of them received a provisional stent (82.0%). Provisionally stented cases underwent spot stenting (24.4%), partial lesion coverage (37.8%), or whole lesion coverage (37.8%) as defined by the operator. The overall mean stent-to-lesion length ratio was 0.7±0.4. The most common reasons for stenting were persistent residual stenosis ≥50% (59.2%) and flow-limiting dissection (53.6%).

**Table 3.**
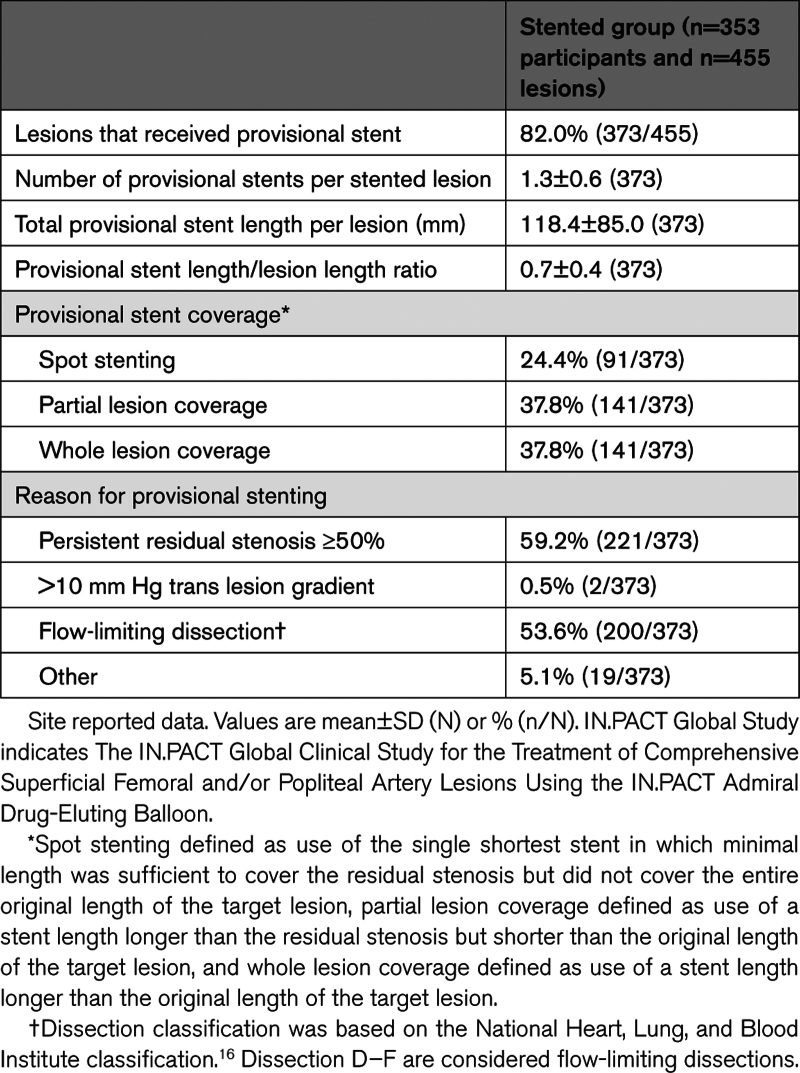
Provisional Stenting in the IN.PACT Global Study

Medication compliance data were available through 36 months and provided in Table S1. The medication compliance rates were not significantly different between stented and nonstented participants.

### Procedural Characteristics

There were significant procedural differences between groups (Table 4). Predilation was performed more frequently in the stented (90.4%) versus nonstented group (73.7%; *P*<0.001), as was postdilation (60.1% stented versus 26.7% nonstented; *P*<0.001). The rates of device, procedural, and clinical success were high across both groups.

**Table 4.**
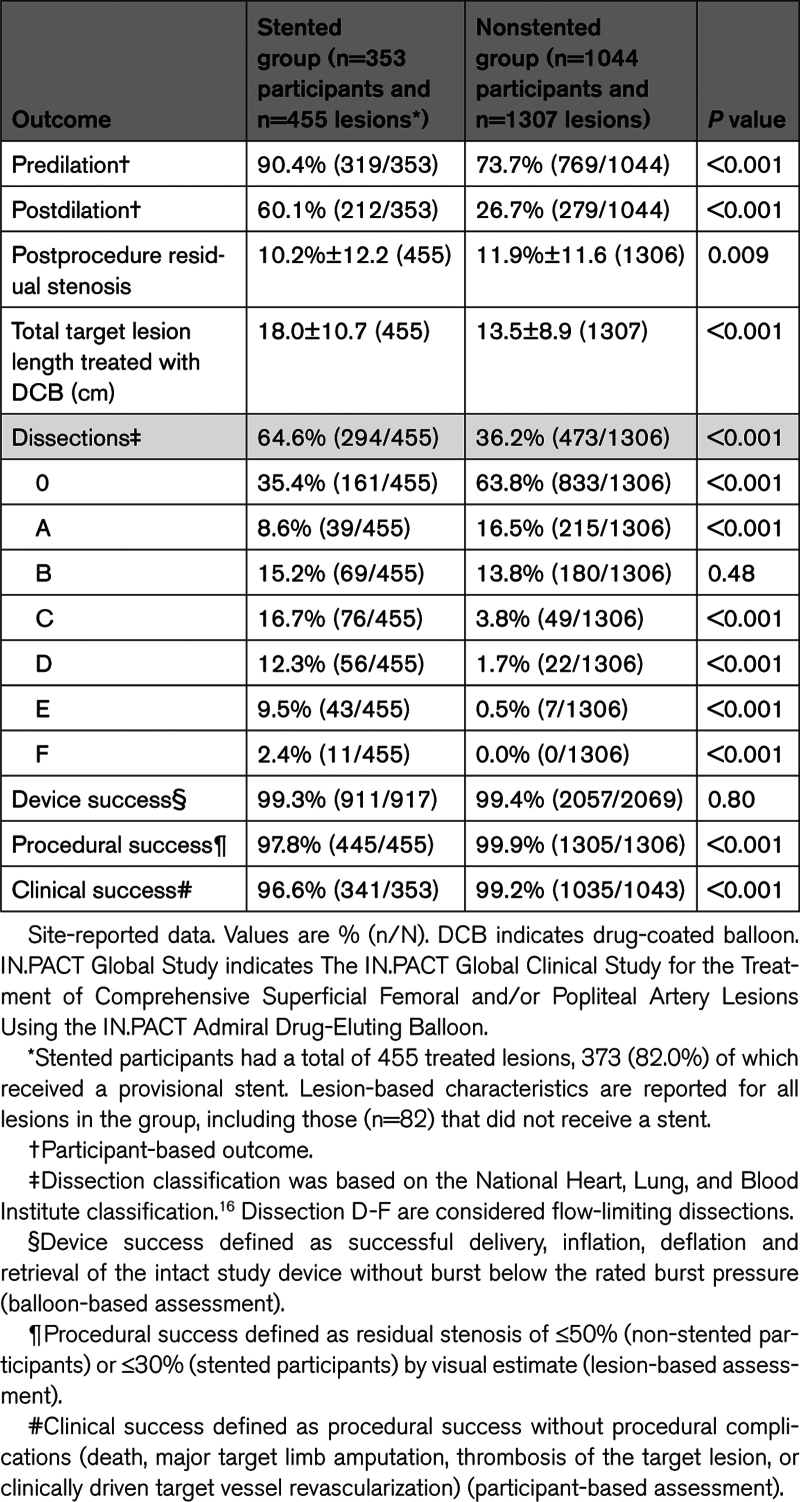
Procedural Outcomes in the IN.PACT Global Study Stented and Nonstented Groups

### Effectiveness Outcomes

**Figure 2.**
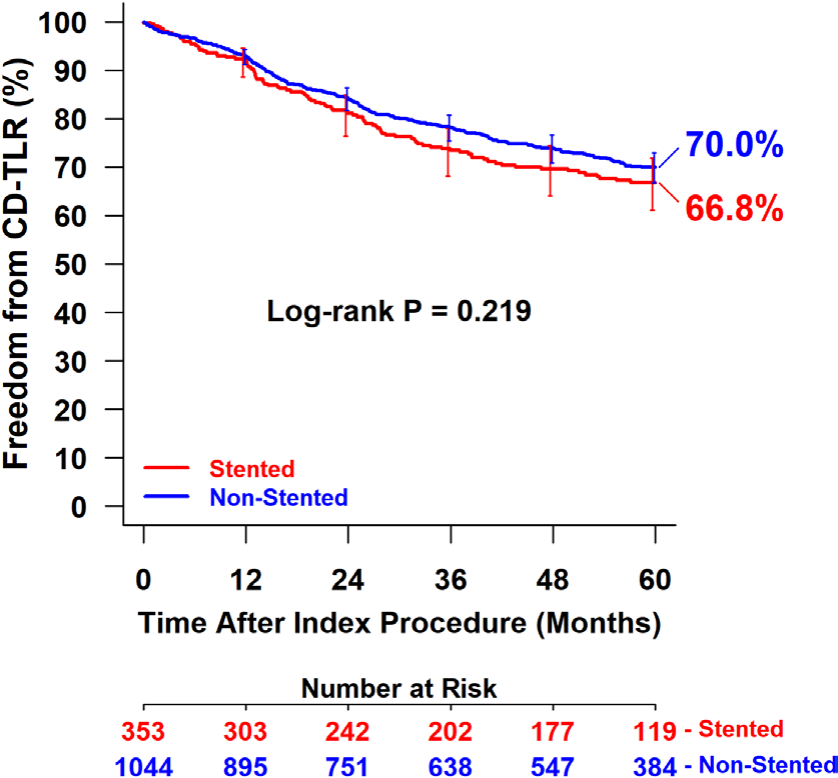
**Kaplan-Meier estimated freedom from clinically driven target lesion revascularization (CD-TLR) through 5 y in the IN.PACT Global Study (The IN.PACT Global Clinical Study for the Treatment of Comprehensive Superficial Femoral and/or Popliteal Artery Lesions Using the IN.PACT Admiral Drug-Eluting Balloon) stented and nonstented groups.** Bars represent 95% CIs. Number at risk represents the number of participants who did not have an event and were not lost to follow-up before the time point.

**Figure 3.**
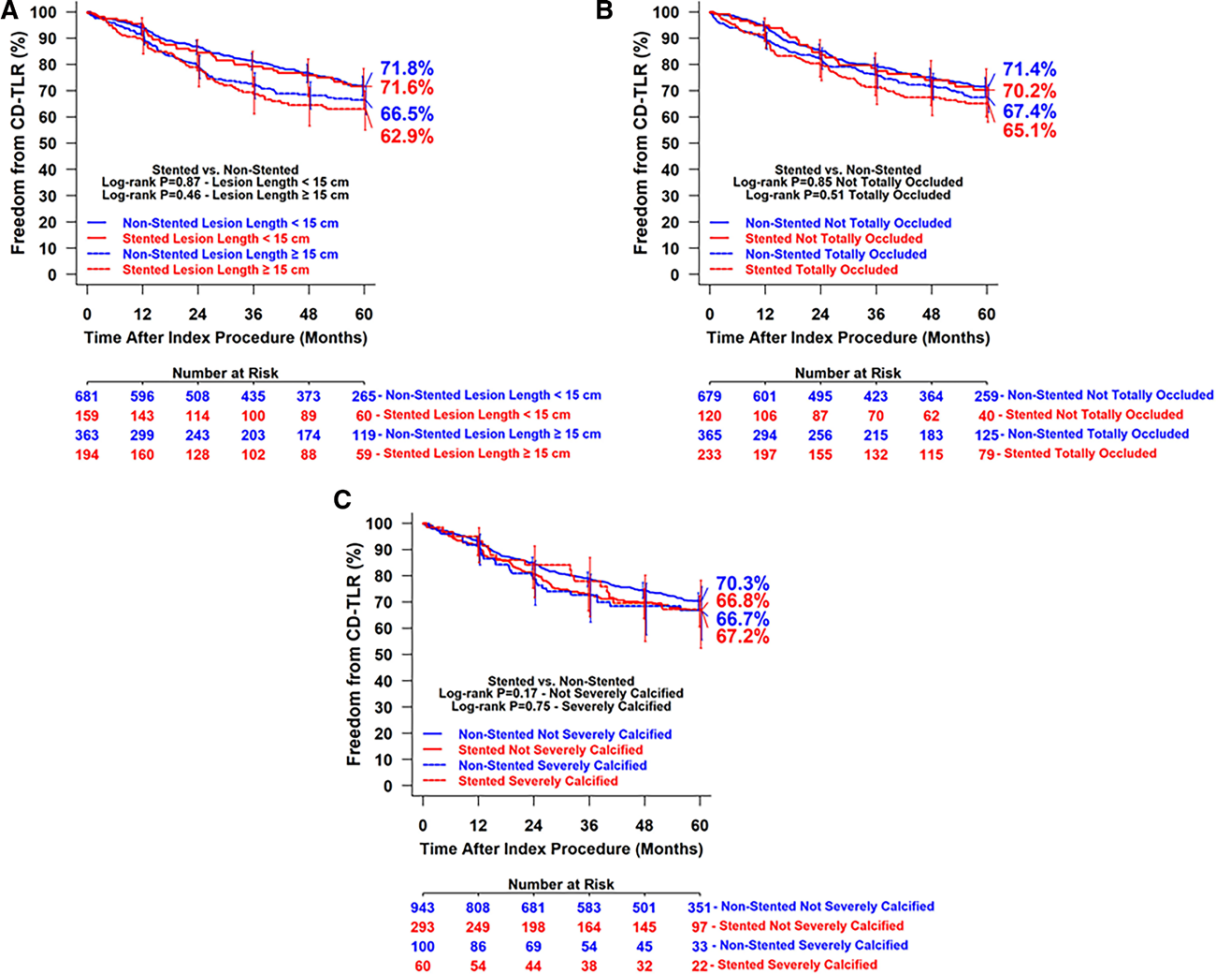
**Subset analyses in the IN.PACT Global Study (The IN.PACT Global Clinical Study for the Treatment of Comprehensive Superficial Femoral and/or Popliteal Artery Lesions Using the IN.PACT Admiral Drug-Eluting Balloon) stented and nonstented groups.** Kaplan-Meier estimated freedom from clinically driven target lesion revascularization (CD-TLR) through 5 y for participants with lesion length ≥15 vs <15 cm (**A**), totally occluded vs not-totally occluded (**B**), and severely calcified vs not-severely calcified (**C**) in the stented and nonstented groups. Bars represent 95% CIs. Number at risk represents the number of participants who did not have an event and were not lost to follow-up before the time point.

Effectiveness outcomes were similar between groups through 5 years (Table 5). Kaplan-Meier estimated freedom from CD-TLR within 5 years was 66.8% for the stented group and 70.0% for the nonstented group (log-rank *P*=0.22; Figure 2). The restricted mean survival time to the first CD-TLR was 1427.9±32.9 days in the stented group and 1481.4±18.1 days in the nonstented group (*P*=0.15). Subset analyses results of freedom from CD-TLR through 5 years by Kaplan-Meier estimate are presented in Figure 3. Participants were stratified by lesion length ≥15 versus <15 cm (Figure 3A); totally occluded versus not-totally occluded (Figure 3B); and severely calcified versus not-severely calcified (Figure 3C) in the stented and nonstented groups. There were no statistical differences between stented and nonstented groups in any of the subsets.

**Table 5.**
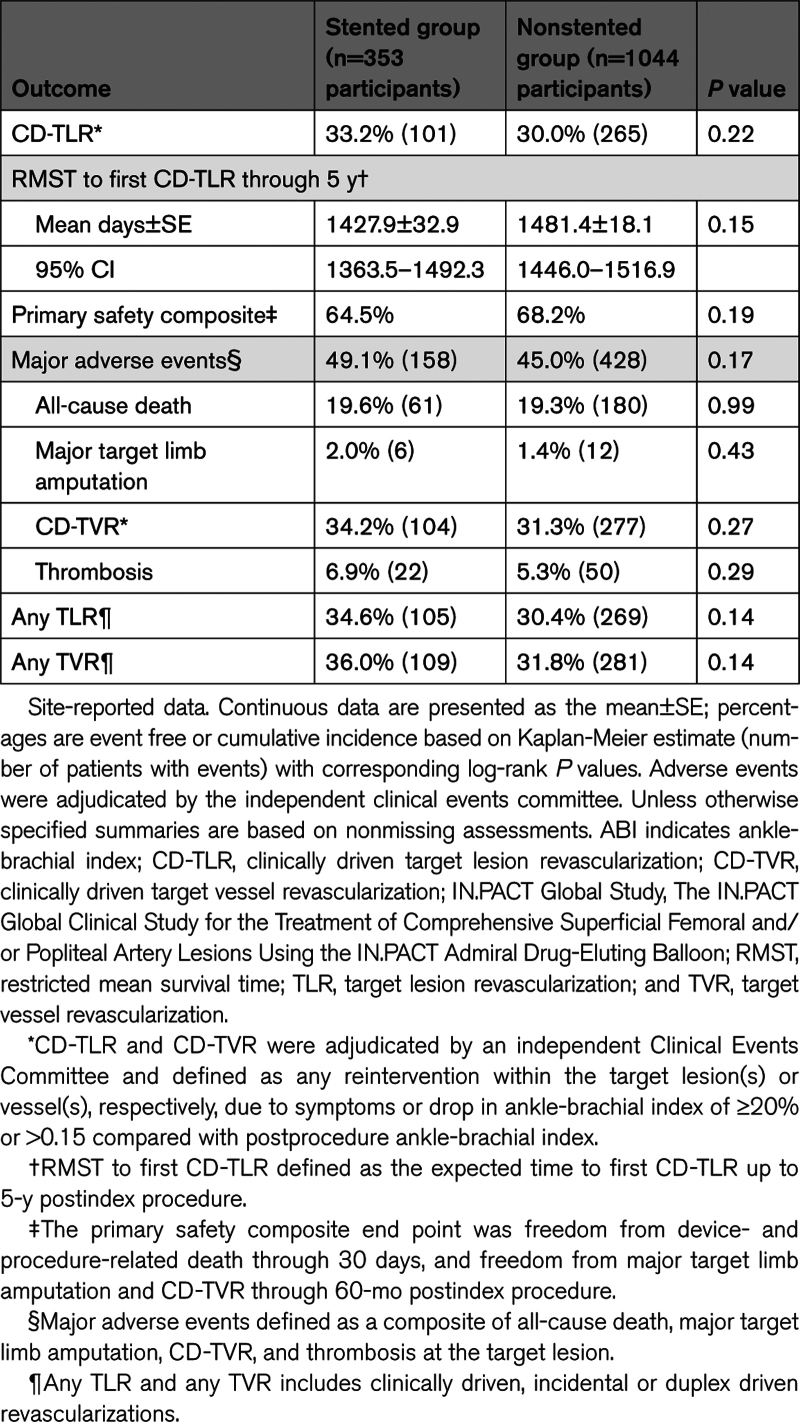
Effectiveness and Safety Outcomes Through 5 Years in the IN.PACT Global Study Stented and Nonstented Groups

### Safety Outcomes

Safety outcomes through 5 years were also similar between groups (Table 5). There was no statistically significant difference between groups in the Kaplan-Meier estimated primary safety composite end point (freedom from device- and procedure-related death through 30 days, and freedom from major target limb amputation and CD-TVR through 60 months; 64.5% stented versus 68.2% nonstented; log-rank *P*=0.19) or the overall cumulative incidence of major adverse events within 5 years (49.1% stented versus 45.0% nonstented; log-rank *P*=0.17). The cumulative incidence of CD-TLR was not different between stented (33.2%) and nonstented groups (30.0%; log-rank *P*=0.22; Table 5). Treating death as a competing risk, the cumulative incidence of CD-TLR was 31.1% in the stented group and 27.7% in the nonstented group and no statistically significant difference was observed (Figure S1). The incidence of major target limb amputations was low in both groups (2.0% stented versus 1.4% nonstented; log-rank *P*=0.43), as was thrombosis (6.9% stented versus 5.3% nonstented; log-rank *P*=0.29).

**Figure 4.**
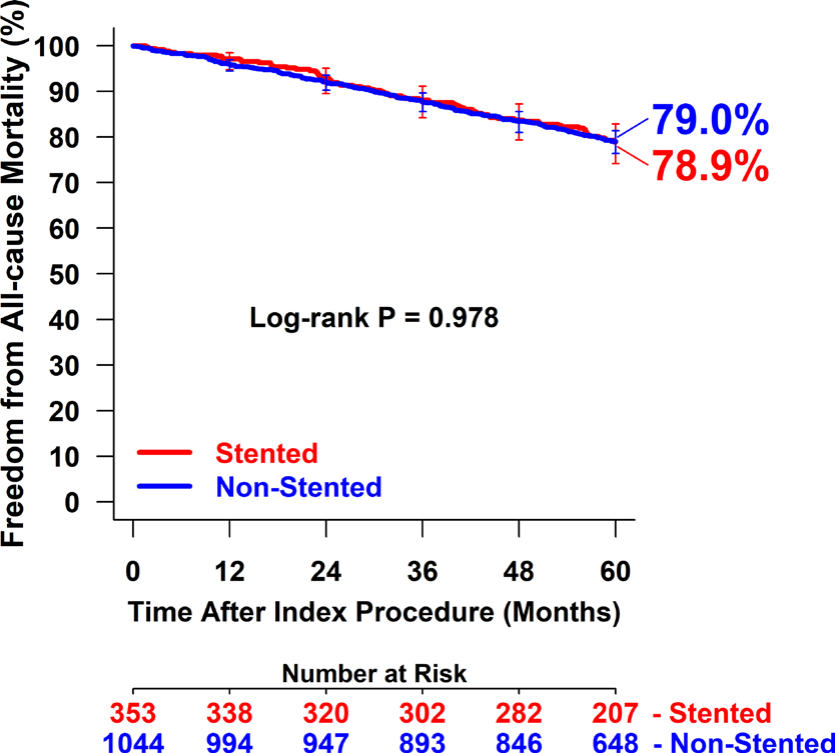
**Kaplan-Meier estimated freedom from all-cause mortality through 5 y (with vital status update) in the IN.PACT Global Study (The IN.PACT Global Clinical Study for the Treatment of Comprehensive Superficial Femoral and/or Popliteal Artery Lesions Using the IN.PACT Admiral Drug-Eluting Balloon) stented and nonstented groups.** Bars represent 95% CIs. Number at risk represents the number of participants who did not have an event and were not lost to follow-up before the time point.

Mortality within 5 years was similar between groups. The cumulative incidence of all-cause death was 19.6% in the stented group and 19.3% in the nonstented group (log-rank *P*=0.99). After the vital status update, 96.4% of participants in the full clinical cohort had vital status information. The Kaplan-Meier estimated freedom from all-cause death based on the vital status update was 78.9% in the stented group and 79.0% in the nonstented group (log-rank *P*=0.98; Figure 4). Deaths identified via vital status collection were not adjudicated by the clinical events committee due to the unavailability of adequate source documentation.

A summary of safety and effectiveness outcomes through 1 to 5 years for stented and nonstented participants from the IN.PACT Global Study based on the final 5-year dataset is reported in Table S2.

Multivariable models were analyzed to adjust for differences in the baseline lesion and clinical characteristics between the stented and nonstented groups for CD-TLR and all-cause death. After adjusting for covariates and accounting for death as a competing risk, the subhazard of CD-TLR was similar in the stented and nonstented participants (subdistribution hazard ratio, 1.02; *P*=0.90; Table S3). After adjusting for covariates, the hazard of all-cause death was also not significantly different between stented and nonstented participants (hazard ratio, 1.03; *P*=0.86; Table S4).

## DISCUSSION

This is the first long-term report from a large, prospective, real-world global study comparing the safety and effectiveness of provisional bare metal stenting after treatment with DCB versus DCB alone for complex femoropopliteal artery lesions in a real-world patient population. Results showed stented and nonstented participants had similar rates of revascularization and safety events, including all-cause death, through 5 years after DCB angioplasty. This is especially notable considering that the stented group had more complex disease, including long lesions, total occlusions, and calcification, than the nonstented group, suggesting that the antirestenotic effect of paclitaxel provided sustained clinical benefit in the provisional bare metal stenting group.

In this study, 25% of participants had DCB followed by provisional stenting to ensure optimal patient outcomes. Provisional bare metal stenting was permitted per protocol if, despite repeated and prolonged balloon inflations, the postprocedure visually estimated residual stenosis was ≥50%, the translesion gradient was >10 mm Hg, or in cases of flow-limiting dissection. Studies have shown that the risk of in stent restenosis is higher with longer stenting especially when involving the popliteal artery.^17^ Encouragingly, most of the stented cases used spot stenting and partial stenting (62.2%), with total lesion coverage in only 37.8%. This is also reflected in the lower overall provisional stent length as compared with the total lesion length (ratio, 0.7). Vessel preparation before DCB use may reduce postprocedure residual stenosis and the need for provisional stenting and may further improve outcomes.^18,19^

In the present study, the majority of the participants (75%) did not need provisional stenting. The 5-year freedom from CD-TLR after DCB was still good in the nonstented group. To our best knowledge, there are no studies that specifically reported DCB outcomes without provisional stenting in real-world populations. Furthermore, contemporary endovascular studies reporting 5-year outcomes of femoropopliteal segments are very limited. Among multicenter femoropopliteal DCB studies, 5-year outcomes were reported by only 3 real-world analyses of the IN.PACT Global Study (clinical cohort, 150-mm DCB cohort and Asian cohort),^20–22^ and 4 RCTs (THUNDER [Local Taxan With Short Time Contact for Reduction of Restenosis in Distal Arteries], IN.PACT SFA [Randomized Trial of IN.PACT (Paclitaxel) Admiral DCB vs Standard Percutaneous Transluminal Angioplasty for the Treatment of Atherosclerotic Lesions in the Superficial Femoral Artery and/or Proximal Popliteal Artery], AcoArt I [Prospective, Multi-center and Randomized Controlled Clinical Study to Verify Effectiveness and Safety of Drug-eluting Balloon in Percutaneous Transluminal Angioplasty Procedure], and EffPac [Multicenter Randomized Controlled Trial to Assess the Effectiveness of Paclitaxel-Coated Luminor Balloon Catheter Versus Uncoated Balloon Catheter in the Superficial Femoral and Popliteal Arteries to Prevent Vessel Restenosis or Reocclusion]).^2–5^ The freedom from CD-TLR rates of 66.8% in the stented and 70.0% in the nonstented groups in the present study were consistent with 69.4% in the IN.PACT Global clinical cohort,^20^ and 72.7% in the IN.PACT Global 150 mm DCB cohort.^21^ As expected, freedom from CD-TLR (66.8% stented and 70.0% nonstented) in the present study was slightly lower than what was reported in the RCTs: THUNDER (79.0% freedom from CD-TLR, mean lesion length 7.4 cm),^2^ IN.PACT SFA (74.5% freedom from CD-TLR, mean lesion length 8.9 cm),^3^ AcoArt I (77.5% freedom from CD-TLR, mean lesion length 14.7 cm),^4^ and EffPac (82.1% freedom from CD-TLR, mean lesion length 5.9 cm).^5^ Interestingly, the IN.PACT Global Asian cohort also had slightly better freedom from CD-TLR (77.1%) compared with the current analysis (66.8% stented and 70.0% nonstented) suggesting some geographic differences.

When compared with drug-eluting stent (DES) outcomes, freedom from CD-TLR through 5 years was 83.1% in the DES arm of the Zilver PTX RCT,^23^ higher than reported in the present study. However, the mean lesion length was much smaller in the Zilver PTX RCT (6.6 cm) compared with the present study (15.4 cm stented and 11.0 cm nonstented). The REAL PTX study (Randomized Evaluation of the Zilver PTX Stent vs Paclitaxel-Eluting Balloons for Treatment of Symptomatic Peripheral Artery Disease of the Femoropopliteal Artery) reported comparable effectiveness and safety outcomes through 1 year for DES versus DCB (bailout stenting as needed), with a favorable patency trend for DES at 36 months.^24^ However, a clinically significant difference was not demonstrated.^24^ Whether there is a difference in outcomes between DES treatment and provisional bare metal stenting after DCB has not been reported.

Long, CTO, and severely calcified lesions are frequently seen in real-world patients but are known to be challenging lesions to treat.^25,26^ Findings from the present study add to the body of evidence supporting the use of paclitaxel DCBs for the full range of lesion types including these challenging subsets in femoropopliteal peripheral artery disease. However, provisional stenting is often required for these types of complex lesions. Previously, the IN.PACT Global Study CTO and long lesion (≥15 cm) cohort analyses showed no difference between stented and nonstented participants in primary patency through 1 year (CTO cohort lesion length: mean 22.8 cm, range, 6.5–53.0 cm; long lesion cohort lesion length: mean 26.4 cm, range, 8.0–47.5 cm).^11,15^ The subset analyses in the present study showed no difference between stented and nonstented groups in freedom from CD-TLR rates through 5 years in participants with lesion length ≥15 cm or participants with total occlusion. Similarly, no differences in 5-year freedom from CD-TLR rates were found in the stented versus nonstented groups with severely calcified lesions. The multivariable regression model further showed that after adjusting for lesion and clinical characteristics that differed at baseline, the risk for CD-TLR remained comparable between the stented and nonstented groups. The increased number of balloon predilations and postdilations in the stented group certainly could have affected patency. However, this was most likely related to the more complex lesions and the attempt at providing a balloon-only result. Postdilation of stents is common so this difference is expected.

There was no difference in the rates of safety events between stented and nonstented participants through 5 years. Major target limb amputation rates were low in both groups (2.0% stented and 1.4% nonstented), and consistent with what has been reported for other paclitaxel DCBs through 5 years (1.4%–2.2%).^27,28^ The cumulative incidence of all-cause mortality in both groups (19.6% stented and 19.3% nonstented) was in line with 5-year mortality data reported for other paclitaxel devices (14.0%–25.9%),^27,29,30^ and was similar or lower than what has been reported at 5 years in epidemiological studies on the natural course of peripheral artery disease (23%–49%).^31–33^ Another important finding of this study was the low thrombosis rate after provisional stenting: 6.9% through 5 years and only 1 episode of late stent thrombosis (between 3 and 5 years). The 5-year incidence of stent thrombosis after endovascular revascularization of the superficial femoral artery was estimated to be around ≈13.4%.^34^ Of note, the evaluation of stent thrombosis will need to be continued outside of this study as very late stent thrombosis occurrence has been reported in the literature.^35^

### Study Limitations

The IN.PACT Global Study is a single-arm trial without a control group. Predefined cohorts (long lesions ≥15 cm, in-stent restenosis, CTO) had core laboratory imaging-based assessments at 1-year postprocedure, but the overall clinical cohort was limited to clinical assessment at all-time points. Similarly, baseline lesion characteristics were reported based on the investigational site reports. Lesion morphology (focal, partial, and diffuse) and stent type were not recorded, which precluded analyses by these variables. Another limitation of this study was that the decision for provisional stenting was at the discretion of the operator and may vary between sites. Because the type of bare metal stent was not captured, the impact of individual stent designs could not be evaluated.

### Conclusions

In this real-world study, revascularization of complex femoropopliteal artery lesions with DCB angioplasty alone or DCB followed by provisional bare metal stenting in certain lesions achieved comparable long-term safety and clinical effectiveness.

## ARTICLE INFORMATION

### Acknowledgments

The authors would like to recognize and thank the participants and investigators involved in the clinical study for their participation. The authors also thank Stefanie Deckers (Medtronic) for research support and Sangeeta Yendrembam (Medtronic) and Zachary Harrelson (Medtronic) for medical writing assistance in accordance with good publication practice guidelines.^36^

### Sources of Funding

The study was sponsored and funded by Medtronic. Authors received no specific funding for preparation of the manuscript.

### Disclosures

Dr Ansel is a consultant for Boston Scientific, Medtronic, Veryan Medical, Cook Medical, Becton Dickinson, Alucent, and Surmodics. Dr Brodmann has received honoraria from Bard Peripheral Vascular, Biotronik, Medtronic, Philips-Spectranetics, and VIVA Physicians. Dr Brodmann is a consultant for Bard Peripheral Vascular, Biotronik, Medtronic, and Philips-Spectranetics. Dr Rocha-Singh is a consultant for Alucent Biomedical and Philips-Spectranetics and received research grant monies from Boston Scientific, Medtronic, and Philips. Mr Menk is a full time employee of Medtronic. Dr Zeller has received honoraria from Abbott Vascular, BIBA Medical, Biotronik, Boston Scientific, Cook Medical, Efemoral, Gore & Associates, Medtronic, Philips-Spectranetics, Shockwave, and Veryan, consulted for Bayer, Boston Scientific, CSI, Gore & Associates, Medtronic, Philips–Spectranetics, Intact Vascular, Shockwave, Veryan, and Vesper Medical Research, and received clinical trial or drug study funds from B. Braun, Bard Peripheral Vascular, Biotronik, Boston Scientific, Cardiovascular Systems, Inc, Cook Medical, Gore & Associates, Intact Vascular, Med Alliance, Medtronic, Philips-Spectranetics, Pluristem, PQ Bypass, Shockwave, Terumo, TriReme, University of Jena, and Veryan, and holds common stock in QT Medical.

### Supplemental Material

Supplemental Methods

Tables S1–S4

Figure S1

## Supplementary Material
