# Supplementary material for: Five-Year Safety and Effectiveness of Paclitaxel Drug-Coated Balloons Alone or With Provisional Bare Metal Stenting for Real-World Femoropopliteal Lesions: IN.PACT Global Study Subgroup Analysis
Source: Circ Cardiovasc Interv. 2024 Feb 13;17(2):e013084. doi: 10.1161/CIRCINTERVENTIONS.123.013084 (PMC10871603; doi:10.1161/CIRCINTERVENTIONS.123.013084)
Supplement: Supplementary file 1 [file hcv-17-e013084-s001.pdf]

SUPPLEMENTAL MATERIAL

Supplemental Content List

- **Supplemental Methods**
- **Table S1.** Medication Compliance by Stented versus Nonstented
- **Table S2.** IN.PACT Global Study Outcomes in Stented (N=353 participants) and Nonstented Groups (N=1044 participants) 1 through 5 Years
- **Table S3.** Subdistribution Hazard Ratios from Fine Gray Model for CD-TLR with Death as a Competing Risk
- **Table S4.** Hazard Ratios from Cox Proportional Hazard Model for All-Cause Death
- **Figure S1.** Clinically driven target lesion revisualization in the presence of death as a competing risk.

SUPPLEMENTAL METHODS

Study Design and Procedure

The IN.PACT Global Study is a prospective, multicenter, international, single-arm clinical study to assess to safety and effectiveness of a paclitaxel-coated drug-coated balloon (DCB) for the treatment of real-world patients with atherosclerotic disease of the femoropopliteal artery. Enrolled participants had symptoms of intermittent claudication and/or ischemic rest pain (Rutherford clinical category 2–4) and angiographic evidence of severe stenosis or occlusion in the femoropopliteal artery, including the entire native superficial femoral artery and/or popliteal artery (P1 to P3). The target lesion had to be ≥2 cm long and could be de novo or

restenotic (in-stent or not in-stent). Due to protocol violations, one Rutherford class 1 participant and 36 Rutherford class 5 participants were enrolled and included in the analysis.

Participants were followed at discharge, 30 days, 12 months and then annually through 60 months. A hospital visit was required at 6, 12, 24 and 36 months while a telephone contact was performed at 30 days, 48 and 60 months. Investigational sites were also asked to obtain vital status from study participants who withdrew or were lost to follow-up to verify safety information. Vital status results are specifically labeled as such when presented.

All participants received the IN.PACT Admiral DCB. Pre-dilation and post-dilation were performed at the discretion of the operator. Provisional stenting with bare metal stents was permitted if one of the following conditions occurred despite repeated and prolonged balloon inflations: visually estimated residual stenosis ≥50%, trans lesion gradient >10 mmHg, or flow-limiting dissection. For categories of provisional stenting, spot stenting was defined as use of the single shortest stent in which minimal length was sufficient to cover the residual stenosis but did not cover the entire original length of the target lesion, partial lesion coverage was defined as use of a stent length longer than the residual stenosis but shorter than the original length of the target lesion, and whole lesion coverage was defined as use of a stent length longer than the original length of the target lesion.

Additional Statistical Analysis

To account for competing risks and adjust for differences in baseline factors between the stented and nonstented groups, a competing risks regression was computed through 1800 days with CD-TLR as the outcome and death as a competing risk. Backward selection was used to

select a more parsimonious model after starting with the model that included stented versus nonstented patient, lesion length (longest lesion length per subject), total occlusion in at least one lesion, age, sex, diabetes, history of coronary artery disease, history of carotid artery disease, history of revascularization, in-stent restenosis in at least one lesion, ankle brachial index (lowest in bilateral patients), at least one lesion with severe calcification, worst case pre-diameter stenosis based on differences observed in clinical and lesion characteristics provided in Table 1 and 2. Variables were removed one at a time from the starting model based on the highest p-value until all p-values were < 0.2 (except for stented versus nonstented, which always remained in the model). Subdistribution hazard ratios are presented.

A Cox proportional hazard model was computed for death through 1800 days to attempt to adjust for differences in the stented versus nonstented groups. For this model, all-cause death without accounting for the vital status update was used. Backward selection was used to estimate a more parsimonious model after starting with the model that included stented versus nonstented patient, lesion length (longest lesion length per subject), total occlusion in at least one lesion, age, sex, diabetes, history of coronary artery disease, history of carotid artery disease, history of revascularization, in-stent restenosis in at least one lesion, ankle brachial index (lowest in bilateral patients), at least one lesion with severe calcification, worst case pre-diameter stenosis based on differences observed in Table 1 and 2. Variables were removed one at a time from the starting model based on the highest p-value until all p-values were < 0.2 (except for stented versus nonstented, which always remained in the model).

Clinical Events Committee

An independent Clinical Events Committee (CEC) (Syntactx, New York, NY) was established to review revascularization events, adjudicate adverse events, and ensure that select end points met protocol-specified criteria. The CEC adjudicated all major adverse events and reviewed all target lesion revascularization and target vessel revascularization events to determine which were clinically driven, defined as any reintervention within the target lesion(s) or vessel(s), respectively, due to symptoms or drop in ankle-brachial index ≥20% or >0.15 compared with post-procedure ankle-brachial index. The CEC included interventional and non-interventional clinicians with relevant expertise who did not participate in the study and did not have conflicts of interest.

Additional Outcomes Definitions

Device success was defined as successful delivery, inflation, deflation, and retrieval of the intact study balloon device without burst below the rated burst pressure. Procedural success was defined as residual stenosis ≤50% for nonstented participants or ≤30% for stented participants by core laboratory assessment (site-reported estimate was used if core laboratory assessment was not available). Clinical success was defined as procedural success without complications (death, major target limb amputation, thrombosis of the target lesion, or TVR prior to discharge). The device success analysis was device (balloon)-based, the procedural success analysis was lesion-based, and the clinical success analysis was participant-based.

Table S1. Medication Compliance by Stented versus Nontented

	Stented (N=353 Participants)	Nontented (N=1044 Participants)	*P* value
**Dual antiplatelet therapy***			
Discharge	92.3% (325/352)	90.6% (943/1041)	0.39
30 Day	79.8% (269/337)	77.4% (772/997)	0.40
6 Months	40.9% (128/313)	40.1% (363/906)	0.84
12 Months	31.6% (94/297)	34.0% (294/865)	0.48
24 Months	25.1% (65/259)	25.6% (193/755)	0.93
36 Months	24.8% (59/238)	24.1% (165/684)	0.86
**Anticoagulants†**			
Discharge	5.7% (20/352)	5.6% (58/1041)	0.89
30 Day	5.9% (20/337)	6.0% (60/997)	1.00
6 Months	7.0% (22/313)	7.7% (70/906)	0.80
12 Months	8.4% (25/297)	7.9% (68/865)	0.80
24 Months	9.3% (24/259)	8.2% (62/755)	0.61
36 Months	8.8% (21/238)	8.8% (60/684)	1.00
**Statins**			
Discharge	70.7% (249/352)	71.7% (746/1041)	0.73
30 Day	69.4% (234/337)	70.8% (706/997)	0.63
6 Months	70.9% (222/313)	71.6% (649/906)	0.83
12 Months	70.7% (210/297)	71.2% (616/865)	0.88
24 Months	68.3% (177/259)	71.1% (537/755)	0.43
36 Months	71.4% (170/238)	70.5% (482/684)	0.81

*Aspirin plus clopidogrel/ticlopidine/prasugrel

†Includes vitamin K antagonists, direct thrombin inhibitors, and other oral anti-coagulants and other (i.e Warfarin, apixaban, betrixaban, dabigatran, edoxaban and rivaroxaban).

Table S2. IN.PACT Global Study Outcomes in Stented (N=353 participants) and Nonstented Groups (N=1044 participants) 1 through 5 Years*†

Follow-up	Group	Freedom from CD-TLR	MAEs‡	All-cause death	Major target limb amputation	CD-TVR	Thrombosis
Year 1	Stented	92.1%	12.7% (44)	2.6% (9)	0.3% (1)	8.5% (29)	3.5% (12)
	Nonstented	92.9%	11.3% (115)	3.8% (39)	0.2% (2)	7.8% (78)	2.5% (25)
Year 2	Stented	81.1%	26.8% (90)	7.0% (23)	0.9% (3)	19.5% (64)	4.8% (16)
	Nonstented	84.2%	23.3% (233)	7.6% (76)	0.5% (5)	16.6% (160)	4.1% (40)
Year 3	Stented	73.4%	36.6% (121)	10.8% (35)	1.3% (4)	27.2% (86)	6.5% (21)
	Nonstented	78.2%	32.1% (315)	11.3% (110)	0.8% (7)	22.6% (212)	5.1% (49)
Year 4	Stented	69.6%	42.6% (139)	15.1% (48)	2.0% (6)	31.0% (96)	6.5% (21)
	Nonstented	73.9%	38.9% (376)	15.3% (146)	0.9% (8)	27.0% (246)	5.1% (49)
Year 5	Stented	66.8%	49.1% (158)	19.6% (61)	2.0% (6)	34.2% (104)	6.9% (22)
	Nonstented	70.0%	45.0% (428)	19.3% (180)	1.4% (12)	31.3% (277)	5.3% (50)

* Summary data was derived using data from the final 5-year dataset

† Percentages are event-free survival or cumulative incidence (number of participants with events) based on Kaplan–Meier estimate.

‡ Major adverse events defined as a composite of all-cause death, major target limb amputation, CD-TVR, and thrombosis at the target lesion and adjudicated an independent Clinical Events Committee. CD-TLR and CD-TVR were defined as any reintervention within the target lesion(s) or vessel(s), respectively, due to symptoms or drop in ABI of ≥20% or >0.15 compared with post-procedure ABI.

CD-TLR, clinically driven target lesion revascularization; CD-TVR, clinically driven target vessel revascularization; and MAEs, major adverse event

Table S3. Subdistribution Hazard Ratios from Fine Gray Model for CD-TLR with Death as a Competing Risk

Parameter	Subdistribution Hazard Ratio	95% Confidence Interval		*P* value
		Lower	Upper	
Stented versus Nonstented	1.02	0.79	1.31	0.90
Lesion length, one centimeter increase*	1.02	1.01	1.04	<0.001
Age, one year older	0.98	0.97	0.99	0.001
Male versus female	0.80	0.64	1.01	0.06
History of coronary artery disease versus none	1.26	1.01	1.57	0.04
History of revascularization versus none	1.20	0.93	1.55	0.15
In-stent restenosis versus no in-stent restenosis†	1.39	1.05	1.84	0.02
Severe calcification versus no severe calcification‡	1.36	0.98	1.87	0.06

*Lesion length was computed as longest lesion per participant

†In-stent restenosis was computed at subject level if any lesion had in-stent restenosis

‡Severe calcification used the worst case in participants with multiple lesions

Table S4. Hazard Ratios from Cox Proportional Hazard Model for All-Cause Death

Parameter	Hazard Ratio	95% Confidence Interval		*P* value
		Lower	Upper	
Stented versus nonstented	1.03	0.75	1.42	0.86
Age, one year increase	1.06	1.04	1.07	<0.001
Diabetes versus no diabetes	1.48	1.11	1.98	0.007
History of coronary artery disease versus none	1.37	1.03	1.83	0.03
History of revascularization versus none	1.35	1.01	1.80	0.045
Ankle brachial index, one unit increase*	0.49	0.25	0.96	0.04

* Ankle brachial index was computed as minimum for bilateral participants

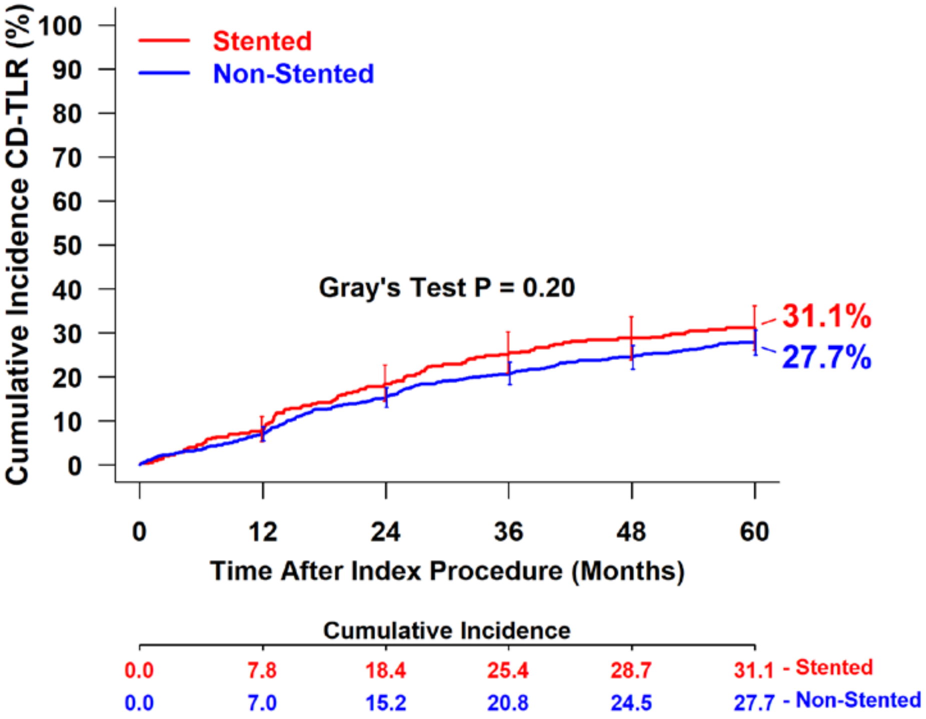

Figure S1: Clinically driven target lesion revascularization (CD-TLR) in the presence of death as a competing risk. Kaplan-Meier estimates of CD-TLR through 5 years were 31.1% and 27.7%, respectively for stented and nonstented groups. The Gray's test p-value comparing two treatment groups through 60 months is 0.20.
